# Surgery for Infective Endocarditis after Primary Transcatheter Aortic-Valve Replacement—A Retrospective Single-Center Analysis

**DOI:** 10.3390/jcm12165177

**Published:** 2023-08-09

**Authors:** Romina Maria Rösch, Lena Brendel, Katja Buschmann, Christian-Friedrich Vahl, Hendrik Treede, Daniel-Sebastian Dohle

**Affiliations:** 1 Department of Thoracic Surgery, Thoraxklinik Heidelberg, Heidelberg University Hospital, 69126 Heidelberg, Germany; lena.brendel@med.uni-heidelberg.de; 2 University Medical Center of the Johannes Gutenberg University Mainz, 55131 Mainz, Germany; katja.buschmann@unimedizin-mainz.de (K.B.); hendrik.treede@unimedizin-mainz.de (H.T.); daniel-sebastian.dohle@unimedizin-mainz.de (D.-S.D.)

**Keywords:** infective endocarditis, prosthetic-valve endocarditis, transcatheter aortic-valve implantation, valve repair, heart-valve prosthesis, heart-valve pathologies, heart-valve treatment

## Abstract

Transcatheter aortic-valve replacement (TAVR) is increasingly being used for the treatment of aortic-valve stenosis. Therefore, the total number of patients with an aortic-valve prosthesis is increasing, causing the incidence of prosthetic-valve endocarditis to increase. Methods: Between March 2016 and July 2019, ten patients underwent surgery due to prosthetic-valve endocarditis after TAVR. They were identified in our institutional database and analyzed. Results: Infective endocarditis was diagnosed 17 ± 16 month after TAVR. Mean age was 79 ± 4.4 years. Microbiological detection showed 6/10 positive blood cultures for enterococcus faecalis. Median EuroScore II was 24.64%. The mean size of the surgically replaced aortic prosthesis was 23.6 ± 1.3 and that of the TAVR was 28.4 ± 2.3 mm. The surgically implanted aortic valves had a mean gradient of 8.5 ± 2.2 mmHg. One patient died in hospital due to septic multiorgan failure. After discharge, all patients survived with a mean follow-up of 9 ± 8 month. Conclusions: With a rising number of patients after TAVR, prosthetic-valve endocarditis will increasingly occur in patients who were previously considered high or intermediate risk. Our results show that patients with TAVR infective endocarditis can be operated on with good results. Surgical therapy should not be withheld from TAVR patients with infective endocarditis.

## 1. Background

Transcatheter aortic-valve replacements (TAVRs) are a “relatively” new method for treating aortic-valve stenosis. In Germany, a total of 22,000 TAVIs were performed in 2019, a 10% increase from the previous year. Worldwide, there are no exact figures, but estimates suggest that about 200,000 TAVIs were performed in 2019.

Transcatheter aortic-valve replacements are effective alternatives to open heart surgery, especially for older patients or those at high surgical risk. The procedures are generally safe and effective, but as with any medical treatment, there are risks and complications that can be associated with transcatheter aortic-valve implantation.

A severe complication may be prosthetic-valve endocarditis (PVE) after transcatheter aortic-valve replacement. Prosthetic-valve endocarditis is a potential life-threatening complication, following transcatheter aortic-valve replacement or surgical aortic-valve replacement (SAVR).

Transcatheter aortic-valve replacement is increasingly being used for the treatment of aortic-valve stenosis [1]. Infective Endocarditis (IE) following transcatheter aortic-valve replacement is a rare but more frequently occurring complication with an incidence up to 3.4% [1,2,3,4,5]. In individual series, even higher incidence rates of 2.3–3.4% per patient-year have been reported [6,7]. Often those patients are treated conservatively because the surgical risk is considered too high.

Like so often in medicine, new techniques bring not only new hopes and advantages, but also new problems. Therefore, the associated long-term complications, including prosthetic endocarditis, should also be considered.

Transcatheter aortic-valve replacement endocarditis diagnosis can be established with valve academic research consortium-2 criteria, which includes the following criteria:

1. Duke (endocarditis) criteria (minor criteria include fever, predisposing cardiac disease, vascular phenomena, immunologic phenomena, and microbiologic and echocardiographic evidence that does not meet the major criteria. The simultaneous presence of the two main criteria allows the clinical diagnosis of definite infective endocarditis to be made).
2. Evidence of abscess, paravalvular leak, pus, or vegetation confirmed by histological or bacteriological studies during a reoperation or (3) abscess, pus, or vegetation confirmed by an autopsy [8].

The most common bacteria causing prosthetic endocarditis and therefore also transcatheter aortic-valve replacement are Staphylococcus aureus, coagulase-negative staphylococci, *Streptococcus viridans*, and *Enterococcus faecalis*. Other bacteria such as *Pseudomonas aeruginosa*, *Escherichia coli*, and *Candida albicans* can also cause prosthetic endocarditis but are less common [9].

The mortality and morbidity of prosthetic endocarditis depend on several factors, including the causative agent, the severity of the infection, the timing of diagnosis and treatment, and the presence of concomitant diseases.

The mortality rate for prosthetic-valve endocarditis is 10% for surgically and 38% for non-surgically treated patients [4]. Mortality is higher in patients with severe concomitant diseases such as diabetes, renal failure, or cancer, and in elderly patients [5,10,11].

The morbidity of prosthetic endocarditis can also be severe and includes complications such as heart failure, stroke, sepsis, emboli, and abscesses. These complications can lead to worsening of the patient’s condition and complicate treatment.

Early diagnosis and treatment of prosthetic endocarditis are important to reduce mortality and morbidity. Early and appropriate antibiotic therapy of the infected prosthesis can help control the infection and prevent complications.

But in many cases, surgical resection remains the only treatment option for prosthetic-valve endocarditis [1].

This study presents the short- and mid-term results of a small series of patients with infective endocarditis after previous transcatheter aortic-valve replacement treated via surgical TAVR explantation and surgical valve replacement [1].

## 2. Methods

### 2.1. Study Design

Transcatheter aortic-valve replacement endocarditis was defined by using the valve academic research consortium-2 criteria. International Classification of Diseases, 10th Revision (ICD-10) codes were used to identify patients in our institutional database who received surgery for endocarditis secondary to transcatheter aortic-valve replacement.

This retrospective single-center data analysis was submitted to and approved by our institutional ethics committee (2020-15008-retrospective).

A total of 10 adult patients underwent surgical aortic-valve replacement for infective endocarditis after transcatheter aortic-valve replacement at our institution between March 2016 and July 2019. The initial implantation of TAVR was performed in only six of the ten cases in our medical center; all other cases were implanted in external medical centers. A total of 1321 TAVR implantations were performed in our center from March 2016 to July 2019.

Clinical status at the time of presentation to our surgical department, demographic data and individual comorbidities, as well as surgical details and postoperative course, were analyzed and evaluated.

### 2.2. Patient Demographics and Cardiovascular Risk Factors

Endocarditis of transcatheter aortic-valve replacement was diagnosed according to the above-mentioned criteria. It was primarily based on echocardiography and, if available, blood cultures. An attending physician was responsible for the evaluation and interpretation of the examinations.

At the time of presentation, the average age was 79 ± 4.4 years. The cohort included seven men and three women. The mean EuroScore II was 24.64 ± 0.3% preoperatively (before surgical aortic valve replacement).

At the time of admission to our surgical department, the following risk factors were present: six (60%) of all patients had chronic obstructive pulmonary disease (COPD), nine (90%) had arterial hypertension, four (40%) had diabetes mellitus type II, three (30%) were smokers at the time of surgery, and one (10%) had coronary heart disease. Furthermore, the following comorbidities could be diagnosed preoperatively: one (10%) had peripheral vascular disease, three (30%) had pulmonary hypertension, seven (70%) had a rhythm irregularity, and six (60%) had a stroke in their medical history (prior to endocarditis). The average left-ventricular ejection fraction was 54 ± 9% (Table S1). The transcatheter aortic-valve replacement models included two Edwards S3, three direct flow valves and two core valves. None of the patients had previously undergone open heart surgery.

### 2.3. Preoperative Details

Prosthetic-valve endocarditis was diagnosed 17 ± 16 month after transcatheter aortic-valve replacement [1]. The following bacteria could be detected microbiologically: 6/10 patients’ blood cultures were positive for enterococcus faecalis, one of each for *Staphylococcus epidermidis*, *Staphylococcus dysgalactiae* and *Streptococcus bovis* [1]. One of the blood cultures remained negative. Indications for reoperation were large floating structures in 8/10 patients, sepsis with hypotension in 5/10, and severe aortic regurgitation in 3/10 patients [1]. The main clinical, echocardiographic, and microbiological findings and complications during infective endocarditis treatment of the cohort are shown in Table S2. The median EuroScore II before surgical aortic-valve replacement was 24.64 ± 0.3%.

### 2.4. Procedural Details

Although some procedural details varied over the period of 3.5 years, the common standards are described:

All patients diagnosed with TAVR endocarditis were admitted to the operating room following preoperative diagnosis. The patients were equipped with at least one arterial pressure line, a central venous access and a large lumen venous access. Cerebral perfusion was monitored by cerebral oximetry. Transesophageal echocardiography was performed routinely. After a median sternotomy, the pericardium was opened and heparin was administered. First arterial followed by venous cannulation was performed with a two-stage cannula. Antegrade cardioplegia via the aortic root was given. After starting the cardiopulmonary bypass (CPB), the patients were cooled. After clamping the aorta and administering the cardioplegic solution, the aorta was incised.

Surgical treatment of TAVR infective endocarditis is challenging in every aspect; explantation, in particular, is a challenging situation.

However, we were able to evaluate one advantage during explantation of the CoreValve^®^. Cooling the flap and surrounding tissue with ice water can be helpful, as Nitinol has the property of deforming when cooled.

After the aortic-valve prosthesis was loosened and removed, the aortic ring was inspected to identify existing abscess cavities.

In cases with abscess formations (Figure S1), the infected tissue was radically debrided, sparing the surrounding structures. This was followed by extensive irrigation of the abscess cavity with saline solution.

The patch was appropriately cut, and sutured in place. After repair, a new prosthetic valve was implanted into the reconstructed annulus.

In all cases, a biological aortic-valve prosthesis was implanted. After rewarming, the patient was weaned from cardiopulmonary bypass. The new aortic-valve prosthesis was checked by transoesophageal echocardiography. Thoracic closure was performed in the common way.

### 2.5. Statistical Analyses

Statistical calculations were performed using GraphPad Prism version 8.3.1 for Mac (GraphPad Software, La Jolla, CA, USA), Wizard Pro data analysis version 1.9.7 (Evan Miller, Chicago, IL, USA), and SPSS 22.0 for Mac (SPSS Inc., Chicago, IL, USA).

Normal assumptions for continuous variables were validated using the Shapiro–Wilk test.

Patient status at time of presentation baseline demographic data and procedural data, as well as short- and long-term survival, were compared using Student’s *t* test.

Variables whose values or distributions differed significantly within the two groups of surviving and deceased patients and appeared clinically meaningful were identified as possible risk factors for early or late death. Survival was assessed using the Kaplan–Meier method and compared using the log-rank test.

All statistical tests were two-sided, with the alpha level for statistical significance set at 0.05. All frequency data are presented as percentages and all continuous data as mean ± standard deviation.

## 3. Results

### 3.1. Procedural Aspects

The mean cardiopulmonary bypass time was 119 ± 45 min with a cross-clamp time of 85 ± 26 min. In five cases, a concomitant procedure was needed due to other underlying cardiac conditions. Concomitant cardiac procedures were mitral-valve repair/replacement (4/10) caused by mitral-valve endocarditis (3/4) and coronary-artery bypass grafting (1/10). Seven patients required annular patch plasty due to a destruction of the annulus (in one case a Manugian patch expansion was performed).

The mean dimension of the surgically replaced aortic-valve prosthesis was 23.6 ± 1.3 and that of the transcatheter aortic-valve replacement was 28.4 ± 2.3 mm. The surgically implanted aortic valves had a mean gradient of 8.5 ± 2.2 mmHg. In three cases, a rapid deployment/seamless aortic valve was implanted.

### 3.2. Outcome and Survival

The median duration of ventilation and length of stay in the intensive-care unit were 20 ± 16 h and 14 ± 16 days. The in-hospital stay duration was 27 ± 25 days, respectively. During the hospital stay, four patients needed temporary dialysis.

One patient died in hospital due to septic multiorgan failure. After discharge, all patients survived with a mean follow-up of 9 ± 8 month [1].

## 4. Discussion

Infective endocarditis following transcatheter aortic-valve replacement is a rare [12] but more frequently occurring complication with an incidence of up to 3.4% [1,2,3,4,5].

Amat-Santos et al. [3] described coagulase-negative staphylococci (24%), enterococci bacteria (21%) and staphylococcus aureus (21%) as the most typical germs causing TAVR infective endocarditis. In our series of ten patients with TAVR infective endocarditis, we identified similar results with enterococci being the most common bacteria.

Infective endocarditis after transcatheter aortic-valve replacement is a rare [12] but more frequently occurring complication with an incidence of up to 3.4% [1,2,3,4,5].

Amat-Santos et al. [3] described coagulase-negative staphylococci (24%), enterococci (21%), and staphylococcus aureus (21%) as the most common pathogens causing infective endocarditis after TAVR. In our series of ten patients with infective endocarditis after TAVR, we found similar results with enterococci as the most common pathogen.

This bacterium is mainly found in the urogenital tract and intestine and leads to bacteremia via infections such as urinary-tract infection or intestinal disease. Other bacteria detected besides that case series include *Staphylococcus epidermidis* (a bacterium found on the surface of the skin), *Staphylococcus dysgalactiae* (also found in the gastrointestinal tract, urogenital tract, and pharynx), and *Streptococcus bovis* (intestinal flora).

A conclusion about the pathway of infection can only be made on the basis of the bacteria detected by blood culture. However, three of the four identified bacteria are found in the digestive tract. Therefore, it can be discussed that special attention should be given to the intestinal health of TAVR patients.

Our high incidence of floating structures demonstrated via echocardiography was similar to those of Amat-Santos et al. [3] and Khalil Ahmad et al. [13]. They most frequently found vegetations at the prosthetic valve.

Because of atypical symptoms at the time of presentation, diagnosis of transcatheter aortic-valve replacement infective endocarditis is difficult. Those variable symptoms might lead to delayed diagnosis of infective endocarditis after transcatheter aortic-valve replacement, resulting in an increased risk of complications of endocarditis. Therefore, precise and early diagnosis is required, which requires timely accurate and high-resolution transesophageal ultrasound examination [8]. Other factors of a delayed diagnosis might be difficult decision making by the treating physicians, transfer from a different hospital, and failure of the initial treatment approach.

Delayed diagnosis and treatment lead to severe prosthetic-valve endocarditis, which often destroys the aortic annulus. Periannular abscesses occur in a significant number of prosthetic-valve endocarditis patients (56–100%) [14,15].

TAVR infective endocarditis is treated conservatively in most cases because these patients are often classified as high-risk patients according to risk scores like the EuroScore II [16]. And even after surgical treatment is indicated, controversy exists about the best timing for surgical therapy.

A randomized trial of patients with native valve infection and relevant valve dysfunction found that early surgery compared with conventional treatment (77% of patients operated on within 48 h) reduced the risk for the composite end points, showing no difference in overall mortality [17]. Nevertheless, the mortality rate for conservative treatment alone is up to 40% [18].

Mangner et al. [19] published the results of a large international observational registry study in March 2022. In this, there was no survival benefit of surgical treatment over antibiotic therapy alone. Nevertheless, surgical treatment should not generally be avoided, a fact also pointed out by Mangner et al. [19] This new patient population will be encountered more frequently in hospital settings in the future and will require individualized decision making [19].

In cases of late diagnosis or ineffective antibiotic therapy, the infection may proceed and lead to more extensive destruction of the aortic periannular abscess [20].

Surgical treatment of TAVR infective endocarditis is challenging in every aspect; explantation, in particular, is a challenging situation. However, there were no significant differences according to prothesis type. In this point of view, our experience did not differ from that of Mangner et al. [19].

In this group of patients, an annular patch plasty had to be performed in seven cases due to annulus destruction. The risk of destruction of the aortic annulus in cases of endocarditis of the prosthetic heart valve has already been described by Okamoto et al. [21].

Furthermore, a similar surgical approach was described by Okamoto et al. [21]. Here, the destroyed aortic annulus was first completely removed from the infected tissue and reconstructed via patch plasty. After successful reconstruction, the aortic valve can then be implanted in the usual procedure.

An alternative in the surgical management of transcatheter aortic-valve replacement endocarditis to the conventional aortic valve may be sutureless valves, which were used in a total of three patients in our case series.

Our experience is similar to those of Roselló-Díez et al. [22]. Due to the more fragile tissue in endocarditis, valve replacement becomes much more difficult. A sutureless prosthesis offers several advantages in this regard. On the one hand, there is less manipulation of the destroyed aortic ring; furthermore, even an attacked aortic ring can be stabilized; last but not least, the duration of surgery can be considerably shortened.

Therefore, surgical therapy should not generally be withheld in transcatheter aortic-valve replacement patients with infective endocarditis. Knowing that, in such cases early surgery can be performed with good results and low in-hospital mortality should be the subject of interdisciplinary discussion in the Heart-Team.

This single-center experience with a sizeable cohort has some limitations, including that it is unclear how long those patients with transcatheter aortic-valve replacement–prosthetic-valve endocarditis were treated with antibiotics preoperatively. Risk factors for adverse outcomes in TAVR-prosthetic-valve endocarditis were not examined here. In addition, the comparison of surgical aortic-valve replacement and transcatheter aortic-valve replacement aortic valves is limited because of the somewhat different size data.

The main objective of this study was to demonstrate that this group of patients can achieve good postoperative outcomes despite the significantly increased perioperative risk and that this group of patients should not be deprived of surgery.

## 5. Conclusions

With the increasing number of patients treated with transcatheter aortic-valve replacement, endocarditis of prosthetic valves is becoming more common.

Not every patient after transcatheter aortic-valve replacement and secondary endocarditis is suitable for surgical therapy. However, the decision should be made in a patient-centered manner by the cardiac team after a detailed risk–benefit assessment and especially after obtaining the patient’s will.

In some cases, surgery remains the only targeted treatment option and can be performed in selected patients with excellent outcomes.

Therefore, surgical therapy should not be rejected a priori in transcatheter aortic-valve replacement patients with infective endocarditis.

## Data Availability

The data presented in this study are available upon request from the corresponding author.

## Supplementary Materials

The following supporting information can be downloaded at: https://www.mdpi.com/article/10.3390/jcm12165177/s1, Figure S1: This figure shows an example of how the infected tissue was radically debrided, after which the abscess cavity was extensively rinsed with saline solution and sealed with a patch. Table S1: Demographics of patients with infectious TAVR endocarditis. Table S2: This table presents the time from implantation of TAVR to diagnosis of TAVR endocarditis, microbiological workup of blood cultures, and if identified, the specific bacterium, as well as Duke criteria corresponding to each patient.

## References

[1] Rösch M.R., Dohle S.D., Brendel L., Buschmann K., Beiras-Fernandez A., Vahl C.-F. (2019). Surgery for Transcatheter Aortic Valve Replacement Endocarditis—A Single-Center Series. Thorac. Cardiovasc. Surg..

[2] Gallouche M., Barone-Rochette G., Pavese P., Bertrand B., Vanzetto G., Bouvaist H., Pierre I., Schmitt D., Fauconnier J., Caspar Y. (2018). Incidence and prevention of infective endocarditis and bacteraemia after transcatheter aortic valve implantation in a French university hospital: A retrospective study. J. Hosp. Infect..

[3] Amat-Santos I.J., Ribeiro H.B., Urena M., Allende R., Houde C., Bedard E., Perron J., DeLarochelliere R., Paradis J.M., Dumont E. (2015). Prosthetic valve endocarditis after transcatheter valve replacement: A systematic review. JACC Cardiovasc. Interv..

[4] Pericas J.M., Llopis J., Cervera C., Sacanella E., Falces C., Andrea R., Garcia de la Maria C., Ninot S., Vidal B., Almela M. (2015). Infective endocarditis in patients with an implanted transcatheter aortic valve: Clinical characteristics and outcome of a new entity. J. Infect..

[5] Regueiro A., Linke A., Latib A., Ihlemann N., Urena M., Walther T., Husser O., Herrmann H.C., Nombela-Franco L., Cheema A.N. (2016). Association Between Transcatheter Aortic Valve Replacement and Subsequent Infective Endocarditis and In-Hospital Death. JAMA.

[6] Lee H.S., Lee S.P., Jung J.H., Kim H.M., Kim C.H., Park J.B., Kim H.K., Kim Y.J., Kim H.S., Sohn D.W. (2014). Infective endocarditis associated with transcatheter aortic valve replacement: Potential importance of local trauma for a deadly nidus. J. Cardiovasc. Ultrasound.

[7] Puls M., Eiffert H., Hunlich M., Schondube F., Hasenfuss G., Seipelt R., Schillinger W. (2013). Prosthetic valve endocarditis after transcatheter aortic valve implantation: The incidence in a single-centre cohort and reflections on clinical, echocardiographic and prognostic features. EuroIntervention.

[8] Kappetein A.P., Head S.J., Genereux P., Piazza N., van Mieghem N.M., Blackstone E.H., Brott T.G., Cohen D.J., Cutlip D.E., van Es G.A. (2012). Updated standardized endpoint definitions for transcatheter aortic valve implantation: The Valve Academic Research Consortium-2 consensus document. Eur. Heart J..

[9] Eisen A., Shapira Y., Sagie A., Kornowski R. (2012). Infective endocarditis in the transcatheter aortic valve replacement era: Comprehensive review of a rare complication. Clin. Cardiol..

[10] Mangner N., Woitek F., Haussig S., Schlotter F., Stachel G., Höllriegel R., Wilde J., Lindner A., Holzhey D., Leontyev S. (2016). Incidence, Predictors, and Outcome of Patients Developing Infective Endocarditis Following Transfemoral Transcatheter Aortic Valve Replacement. J. Am. Coll. Cardiol..

[11] Del Val D., Linke A., Abdel-Wahab M., Latib A., Ihlemann N., Urena M., Won-Keun K., Husser O., Herrmann H.C., Nombela-Franco L. (2020). Long-Term Outcomes After Infective Endocarditis After Transcatheter Aortic Valve Replacement. Circulation.

[12] Carnero-Alcazar M., Maroto Castellanos C.L., Carnicer J.C., Rodriguez Hernandez J.E. (2010). Transapical aortic valve prosthetic endocarditis. Interact. Cardiovasc. Thorac. Surg..

[13] Khalil Ahmad K.E.K., Hjortdal V., Nørgaard B.L., Terkelsen C.J., Jensen K., Christiansen E.C., Terp K.A., Andersen G., Hvitfeldt S., Andersen H.R. (2016). Prosthetic valve endocarditis after transcatheter aortic valve implantation-diagnostic and surgical considerations. J. Thorac. Dis..

[14] Baumgartner F.J., Omari B.O., Robertson J.M., Nelson R.J., Pandya A., Pandya A., Milliken J.C. (2000). Annular abscesses in surgical endocarditis: Anatomic, clinical, and operative features. Ann. Thorac. Surg..

[15] Bayer A.S., Bolger A.F., Taubert K.A., Wilson W., Steckelberg J., Karchmer A.W., Levison M., Chambers H.F., Dajani A.S., Gewitz M.H. (1998). Diagnosis and management of infective endocarditis and its complications. Circulation.

[16] Castiglioni A., Pozzoli A., Maisano F., Alfieri O. (2012). Endocarditis after transfemoral aortic valve implantation in a patient with Osler-Weber-Rendu syndrome. Interact. Cardiovasc. Thorac. Surg..

[17] Kang D.H., Kim Y.J., Kim S.H., Sun B.J., Kim D.H., Yun S.C., Song J.M., Choo S.J., Chung C.H., Song J.K. (2012). Early surgery versus conventional treatment for infective endocarditis. N. Engl. J. Med..

[18] Wong D.R., Boone R.H., Thompson C.R., Allard M.F., Altwegg L., Carere R.G., Cheung A., Ye J., Lichtenstein S.V., Ling H. (2009). Mitral valve injury late after transcatheter aortic valve implantation. J. Thorac. Cardiovasc. Surg..

[19] Mangner N., del Val D., Abdel-Wahab M., Crusius L., Durand E., Ihlemann N., Urena M., Pellegrini C., Giannini F., Gasior T. (2022). Surgical Treatment of Patients With Infective Endocarditis After Transcatheter Aortic Valve Implantation. J. Am. Coll. Cardiol..

[20] Okada K., Okita Y. (2013). Surgical treatment for aortic periannular abscess/pseudoaneurysm caused by infective endocarditis. Gen. Thorac. Cardiovasc. Surg..

[21] Okamoto Y., Minakata K., Yunoki T., Katsu M., Chino S.-i., Matsumoto M. (2011). Treatment of prosthetic valve endocarditis complicated by destruction of the aortic annulus. Gen. Thorac. Cardiovasc. Surg..

[22] Roselló-Díez E., Cuerpo G., Estévez F., Muñoz-Guijosa C., Tauron M., Cuenca J.J., González-Pinto Á., Padró J.M. (2018). Use of the Perceval Sutureless Valve in Active Prosthetic Aortic Valve Endocarditis. Ann. Thorac. Surg..

