# Supplementary material for: Surgery for Infective Endocarditis after Primary Transcatheter Aortic-Valve Replacement—A Retrospective Single-Center Analysis
Source: J Clin Med. 2023 Aug 9;12(16):5177. doi: 10.3390/jcm12165177 (PMC10456027; doi:10.3390/jcm12165177)
Supplement: Supplementary file 1 [file jcm-12-05177-s001.zip › Table S1.pdf]

Table S1. Demographics of patients with infectious TAVR endocarditis.

	Mean ± SD<br>N (%)
**Demographics**	
Age (years)	79 ± 4.4
Gender (Male)	7 (70)
BMI	32 ± 9
EuroScore II	24.64 ± 0.3
**Risk factors**	
COPD	6 (60)
aHTN	9 (90)
Diabetes	4 (40)
Smoking	3 (30)
CAD	1 (10)
**Comorbidities**	
PVD	1 (10)
pHTN	3 (30)
Sinus Rhythm	3 (30)
Previous Stroke	6 (60)
LVEF	54 ± 9
**Procedural Parameters**	
CPB time (min)	119 ± 45
X-clamping time (min)	85 ± 26
Annular patch plasty	8 (80)
Sutureless valve	3 (30)
Concomitant CABG	1 (10)
Concomitant Mitral	4 (40)
