# Supplementary material for: Surgery for Infective Endocarditis after Primary Transcatheter Aortic-Valve Replacement—A Retrospective Single-Center Analysis
Source: J Clin Med. 2023 Aug 9;12(16):5177. doi: 10.3390/jcm12165177 (PMC10456027; doi:10.3390/jcm12165177)
Supplement: Supplementary file 1 [file jcm-12-05177-s001.zip › Table S2.pdf]

Table S2: This table presents the time from implantation of TAVR to diagnosis of TAVR endocarditis, microbiological workup of blood cultures, and if identified, the specific bacterium, as well as Duke criteria corresponding to each patient.

Patient	Time since implantation (m)	Bacteria	Other Duke-criteria
1	1	Stap. epi.	PVL, Stroke
2	38	Enter. faecalis	Vegetation, Stroke
3	13	Stap. dysgalac.	Vegetation, PVL, Embolism
4	22	Enter. faecalis	PVL
5	4	Enter. faecalis	Vegetation, Embolism
6	8	Enter. faecalis	Vegetation, Embolism
7	9	Enter. faecalis	Vegetation
8	18	Enter. faecalis	Vegetation
9	53	Strep. bovis	Vegetation
10	0.25	-	Vegetation
