# Supplementary figures and images for: Surgery for Infective Endocarditis after Primary Transcatheter Aortic-Valve Replacement—A Retrospective Single-Center Analysis
Source: J Clin Med. 2023 Aug 9;12(16):5177. doi: 10.3390/jcm12165177 (PMC10456027; doi:10.3390/jcm12165177)

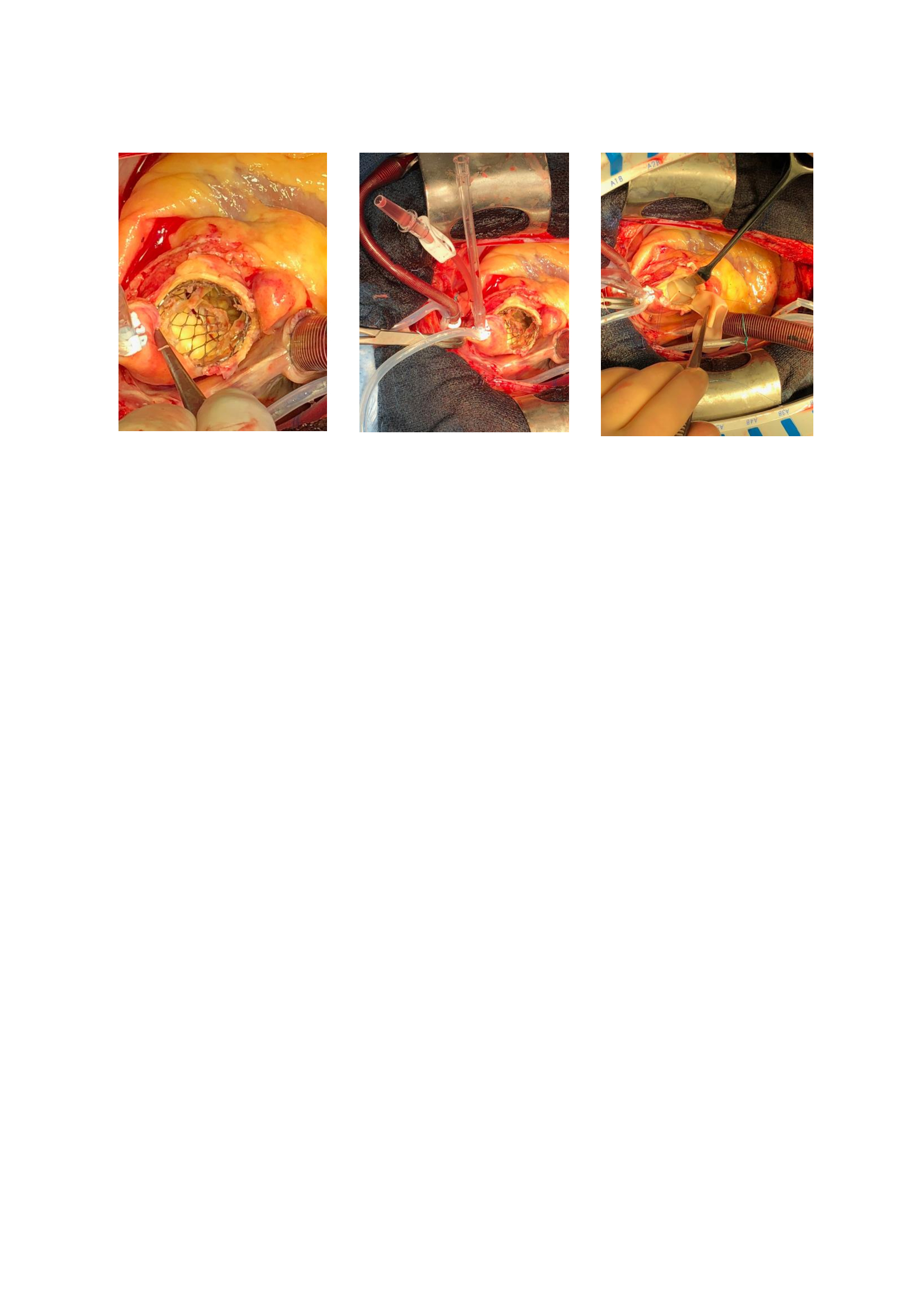

Supplement: Supplementary file 1 [file jcm-12-05177-s001.zip › Figure S1.jpg]
